# Strengths, limitations, and way forward of home-based rehabilitation practices after stroke: a scoping review

**DOI:** 10.1186/s12913-026-14139-4

**Published:** 2026-02-25

**Authors:** Luca Oppici, Ann Marie Hestetun-Mandrup, Matheus M. Pacheco, Emilie Halmrast Simonsen, Arve Opheim, Lena Rafsten, Marianne Løvstad, Guna Bērziņa, Katharina S. Sunnerhagen, James R. Rudd

**Affiliations:** 1 The Department of Teacher Education and Outdoor Studies, Norwegian School of Sport Sciences, Oslo, 0863 Norway; 2 Monash University European Research Foundation ETS, Prato, Italy; 3 Center for Research and Education, Sunnaas Rehabilitation Hospital, Nesoddtangen, 1450 Norway; 4 Department of Rehabilitation Science and Health Technology, Oslo Metropolitan University, Oslo, Norway; 5 University of São Paulo, School of Physical Education and Sport, São Paulo, Brazil; 6 CIFI2D, Faculty of Sport, University of Porto, Porto, Portugal; 7 Department of Clinical Neuroscience and Rehabilitation Medicine, Institute of Neuroscience and Physiology, Sahlgrenska Academy, University of Gothenburg, Gothenburg, Sweden; 8 Department of Occupational Therapy and Physiotherapy, Sahlgrenska University Hospital, Gothenburg, Sweden; 9 Department of Psychology, Faculty of Social Sciences, University of Oslo, Oslo, Norway; 10 Department of Rehabilitation, Faculty of Health and Sport Sciences, Riga Stradiņš University, Riga, Latvia; 11 Clinic of Rehabilitation, Riga East University Hospital, Riga, Latvia; 12 Rehabilitation Medicine, Sahlgrenska University Hospital, Gothenburg, Sweden; 13 Department of Sport, Food and Natural Sciences, Faculty of Education, Arts and Sports, Western Norway University of Applied Sciences, Sogndal, 6856 Norway

**Keywords:** Physiotherapy, Therapy, Home-based, Pedagogy, Task-specific, Context-specific, Stroke, Motor rehabilitation

## Abstract

**Background:**

Home-based motor rehabilitation after stroke has a great potential to promote task-specific and context-specific training in a familiar, functionally rich context, supporting more personalised, engaging, and adaptable rehabilitation experiences. This review aims to map existing practices in home-based motor rehabilitation after stroke and critically examine whether this potential is fully utilized and identify strengths and limitations. It further explores how healthcare professionals can optimise their planning and delivery of interventions.

**Methods:**

A scoping review was conducted following the PRISMA guidelines. Comprehensive searches were conducted in 5 databases: PubMed, CINAHL, MEDLINE, APA PsycINFO and Web of Science. Two reviewers independently screened the studies for eligibility and extracted characteristics of each study into a data charting table.

**Results:**

Sixty-six studies met the inclusion criteria. The results highlighted a consistent implementation of task-specific exercises but limited implementation of context-specific stimuli, inadequate training dosage, and limited contextual adaptation to the home environment. Further, instructional methods tended to be overly prescriptive, co-design strategies applied inconsistently, and caregiver engagement often underutilised.

**Conclusions:**

Existing practices do not fully utilize the potential of home-based rehabilitation. We argue clinical work should prioritise early-phase interventions with adequate intensity and develop pedagogical frameworks to guide home modifications and instructional methods. The framework should support progressive and engaging practice, and fully adopt co-designed, person-centred approaches that meaningfully involve family caregivers.

**Supplementary Information:**

The online version contains supplementary material available at 10.1186/s12913-026-14139-4.

## Background

Stroke is one of the leading causes of death and adult disability worldwide, representing a serious and disabling global health-care challenge [1, 2]. While most people survive the initial stroke event, approximately 80% are left with motor impairments, which manifest in a functional impairment of muscle control, movement or mobility [3]. Motor rehabilitation after stroke aims to reduce these impairments and improve movement functions and independence in activities of daily living (ADL) [4].

With a widespread emphasis on early hospital discharge, there is a growing interest and need of conducting rehabilitation after stroke directly in the people’s home [5]. Initially considered only as a cost-effective alternative to hospital-based care, home-based rehabilitation can offer additional benefits beyond the financial considerations. The home environment has in fact the potential to provide a familiar, functionally rich context that can support personalised, engaging, and adaptable rehabilitation experiences. When well designed, home-based training can deliver appropriate intensity and frequency, while enabling repetitive, goal-oriented, progressive, and context-specific practice.

One of the key strengths of home-based rehabilitation is the possibility to promote task-specific and context-specific training. Motor learning research consistently shows that effective skill transfer occurs when the training environment closely mirrors the real-world context in which the skill is used [6, 7]. The home naturally offers a wide array of stimuli for meaningful and functional – task- and context-specific – movement practice. For example, the task of reaching and grasping can be performed repeatedly using everyday objects of varied size, texture, and weight ranging from cups, towels, and jars. This variability of stimuli can foster motor adaptation and supports the development of flexible, robust movement solutions tailored to the individual’s functional needs [8]. Furthermore, instructional methods grounded in motor learning theory can further enhance the benefits of home-based rehabilitation. Instructions and feedback can be tailored to each individual, task and context to promote active problem-solving and fine-tune variability in practice, aligning with the complex and dynamic nature of stroke recovery [9, 10].

Beyond the physical development potential, home-based rehabilitation has the potential to support greater autonomy and motivation. Since healthcare professionals often supervise patients only for limited periods, self-directed and spouse-supported practice becomes essential. Maintaining consistent and effective engagement requires patients to be motivated, confident, and empowered to take ownership of their rehabilitation. A person-centred approach, grounded in co-design and aligned with individual goals can improve motivation. Involving patients and their family members in the design and delivery of rehabilitation tasks has been shown to increase adherence, enhance relevance, and support long-term sustainability [11, 12].

Although home-based rehabilitation following stroke holds significant promise, it remains unclear how the home environment is currently utilised to support motor recovery, and whether established principles of motor learning and rehabilitation are being effectively applied. The European Stroke Action Plan suggests that the potential of home-based rehabilitation is unrealised [13]. To address this gap, this scoping review maps existing practices in home-based motor rehabilitation after stroke. Specifically, it examines training dose, training specificity, the use of the home environment, teaching practices, and intervention design. Furthermore, the review critically explores how healthcare professionals can integrate and optimise these elements in their planning and delivery of home-based motor rehabilitation to better support stroke survivors.

## Methods

The current study follows the guidelines proposed by the Preferred Reporting Items for Systematic reviews and Meta-Analyses extension for Scoping Reviews (PRISMA-ScR [14], see the PRISMA checklist). This scoping review was not pre-registered.

### Eligibility criteria

Considering that this review does not aim to synthesize the effectiveness of an intervention, the eligibility criteria contain only the Population and Intervention of the PICOS statement: Population: stroke survivors; Intervention: home-based motor rehabilitation led by a healthcare professional (see the full list of inclusion and exclusion criteria in additional file 1).

### Information sources and search strategy

A comprehensive literature search was conducted from the inception of literature to the search date in: PubMed, CINAHL, MEDLINE, APA PsycINFO and Web of Science. The search was initially performed on the 6th of September 2024 and updated on the 11th of August 2025. The reference list of the studies included and relevant review articles [5, 11, 15–20] were screened to identify additional relevant studies.

The search terms included the domains of “home” (Home or home-based or home based or home environment or residential or domiciliary or home-delivered or domestic or home-centred or home-centred), “rehabilitation” (rehabilitation or treatment or rehabilitative or therapy or recovery or “task specific training” or “task oriented training”), “therapist” (therapist or therapist-led or therapist led or care-supported or care supported or physiotherapist or nurse or specialist), and “study design” (trial or pilot or experiment or intervention), with the exclusion of systematic reviews (see the search strings in additional file 2).

### Selection process

All records identified through the searches were exported into Endnote X9 software (Clarivate, Philadelphia, USA), where duplicates were removed automatically and checked manually for unrecognized duplicates. Titles and abstracts were screened first, and then the full-text articles were screened to determine final eligibility. Two authors (ES and LO) independently conducted the screening process, cross-checked their results, and resolved their disagreements in a meeting. If disagreements were not resolved, a third author (JR) was involved to finally decide on the eligibility of studies.

### Data extraction

The following data was extracted from each study: (1) *general information* (author, year, country, study design), (2) *sample characteristics* (sample size, age, gender, time since stroke), (3) *type and focus of intervention*,* and targeted impairment* (based on intervention categories [3] and the studies’ primary outcomes, following the World Health Organization International Classification of Functioning, Disability, and Health Framework [21]), (4) *intervention dose* (duration, planned weekly dose, completed weekly dose, repetitions per session), (5) *supervision* (healthcare profession, modality, frequency, supervised sessions), (6) *training specificity* (whether the exercises were task-specific and context-specific), (7) *use of the home environment* (whether modifications were made to the home environment, and whether different aspects of the home were used as stimuli), (8) *teaching practices* (prescriptive or open-ended instruction and feedback), (9) *intervention design* (co-designed or therapist-designed), (10) *individualization of the intervention* (individualized or one-size-fits all), 11) *progression of tasks and challenge* (individualized or predefined); and 12) *involvement of family/caregiver*. See Table 1 for details of the operationalization of these categories. Information on intervention outcomes was not extracted as this scoping review is not concerned with the effectiveness of the trials.

**Table 1:** Operationalization of the key information extracted from each study

Category	Coding
**General information** (author, year, country, study design)	Directly provided in the study
**Sample characteristics** (sample size, age, gender, time since stroke)	Directly provided in the study
**Type and focus of intervention**,** and targeted impairment**	Directly provided in the study and categorised following WHO International Classification of Functioning, Disability, and Health Framework
**Intervention dose** (duration, planned weekly dose, completed weekly dose, repetitions per session)	Directly provided in the study
**Supervision** (healthcare profession, modality, frequency, supervised sessions)	Directly provided in the study
**Training specificity** (whether the exercises were task-specific and context-specific)	Task specific: when exercises and movements were specific to functional tasks, such as reach and grasp, walk, go up and down the stairs, and mop the floor Context specific: when exercises and movements were performed in the specific context they are normally performed and using the contextual elements of daily life in the home, such as reaching and grasping cups from kitchen cupboards, mopping the kitchen and bathroom floor, and going up and down the stairs
**Use of the home environment** (whether modifications were made to the home environment, and whether different aspects of the home were used as stimuli)	Modifications of the home: directly provided in the study Different aspects of the home used as stimuli: when explicitly stated that stroke survivors interacted with different aspects of the house during their exercises
**Teaching practices** (prescriptive or open-ended instruction and feedback)	Prescriptive: when instructions and feedback pointed towards an ideal or preferred movement Open ended: when they did not direct patients towards the ideal movement but promoted the discovery of the movements that best fit them
**Intervention design** (co-designed or therapist-designed)	Co-designed: when stroke survivors were explicitly included in the design process Therapist-designed: when the therapist designed the intervention without consulting the stroke survivor
**Individualization of the intervention** (individualized or one-size-fits all)	Individualized: when the rehabilitation exercises were tailored to the condition, needs, abilities and goals of each individual person One-size-fits-all: when exercises were prescribed generally and not tailored to each person
**Progression of tasks and challenge** (individualized or predefined)	Directly provided in the study
**Involvement of family/caregiver**	Directly provided in the study
When the study description did not provide enough information to infer a specific aspect, it was coded as “not described”	

One author (LO) extracted the data, and another author (ES) assessed the accuracy of the extracted data.

### Synthesis of results

According to the aim of this review, a descriptive synthesis of the strategies employed by therapists in home-based rehabilitation was performed.

## Results

### Study selection

**Fig. 1:**
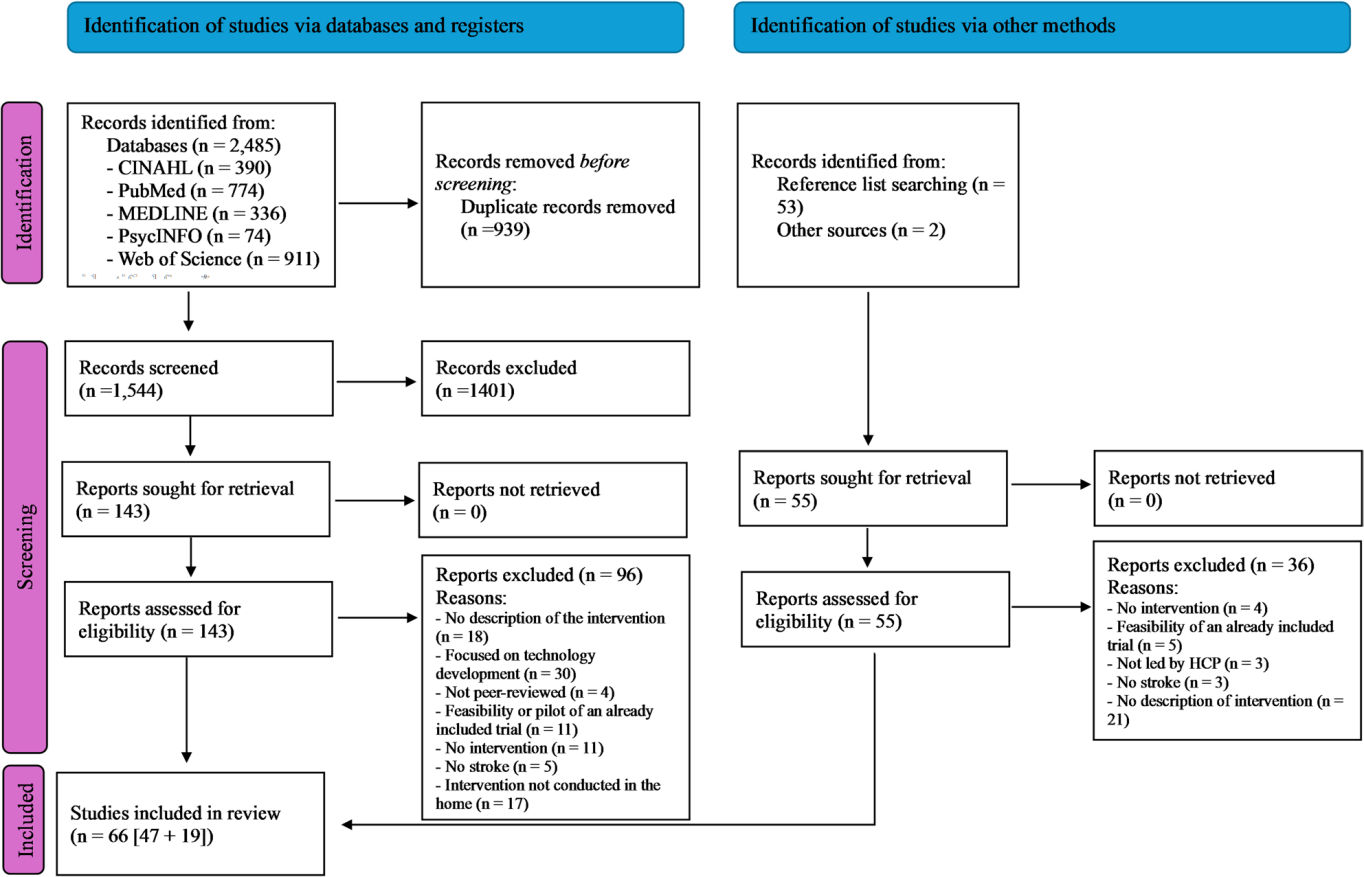
PRISMA flow diagram of the screening process [105]

The initial search through the databases identified 2485 studies (CINAHL, *n* = 390; MEDLINE, *n* = 336; PsycINFO, *n* = 74; Web of Science, *n* = 911; PubMed, *n* = 774). After the removal of 939 duplicates, 1401 studies were excluded based on their title and abstract. The full texts of 143 studies were screened, and 47 studies met the inclusion criteria. The additional screening of references and reviews identified 55 relevant studies, 19 of which met the inclusion criteria. A total of 66 studies were included in the review (Fig. 1).

### Study characteristics

The included studies involved a total of 2933 participants in the intervention group with a mean age of 65 years and 46% of females on average. Only 58% of studies reported the participants’ time since stroke, which averaged to 23 months. Thirty-seven studies adopted a randomized-controlled design [22–58], three studies a non-randomized controlled design [59–61], eleven studies a one group pre-post design [62–72], three studies a crossover design [73–75], two were case studies [76, 77], and ten studies were protocols [78–87]. The details of each study and the aggregated data relevant to the next sections are presented in the supplementary material.

### Synthesis of results

#### Intervention type, focus, dose, supervision

Details on intervention type, focus, dose are provided in Table 2. The most common intervention types were ADL (17%), physical fitness (14%), and neurophysiological (14%) training. The focus and primary outcomes of the interventions were primarily on activity (47%), function (30%), and both function and activity (21%). The interventions targeted primarily both upper and lower limbs (47%), followed by upper limbs (35%) and lower limbs (17%).

While all studies reported the duration of their intervention (12 weeks on average), information was not always reported for the planned and completed weekly dose, and repetitions per session. Only 60% of studies reported information on the planned weekly dose, which was on average 287 min, and for 40% of the studies the completed weekly dose averaged to 173 min. Only 14% of the studies directly reported the completed weekly dose, for the other studies the completed weekly dose was inferred from the planned weekly dose when all the sessions were supervised by the healthcare professional. For the studies that reported both planned and completed weekly dose (13% of studies), the ratio of completion rate was on average 93%. Lastly, only 9% of studies reported the number of repetitions per session (175 on average). This high number of repetitions reported was related to the adopted repetitive-task training, which entails a high number of task repetitions.

**Table 2:** Aggregated data on intervention type and focus, targeted impairment, and training dose

Intervention			Dose		
Type	Focus	Targeted impairment	Duration in weeks: Mean (SD)	Planned weekly dose in minutes: Mean (SD)	Completed weekly dose in minutes: Mean (SD)
CIMT: 9% ADL: 17% NP: 14% ML: 9% PFT: 14% HIT: 2% RTT: 8% NP, ADL: 9% NP, RTT: 2% PFT, ML: 5% NP, ML: 9% RTT, ML: 5%	F: 30% A: 47% F, A: 21% P: 2%	UL: 35% LL: 17% UL, LL: 47%	12 (13,5)	287 (233) ^1^	173 (94) ^2^

Note: CIMT (constrained-induced movement therapy), ADL (activities of daily living), NP (neurophysiological), ML (motor learning), PFT (physical fitness training), HIT (high-intensity training), RTT (repetitive task training), F (function), A (activity), P (participation), UL (upper limb), LL (lower limb)

^1^ 60% of studies reported this information; ^2^ 40% of studies reported this information

Details on supervision are provided in Table 3. The interventions were supervised primarily by physiotherapists (46%), occupational therapists (21%), a combination of physiotherapists and occupational therapists (13%), or others. Supervision was primarily conducted in presence, approximately twice a week on average, with an average supervision of 66% of the total number of sessions. In 20 out of 64 studies, all sessions were supervised by a healthcare professional. For the studies (8%) where not all the sessions were supervised, the rate of weekly dose completion was on average 93%.

**Table 3:** Aggregated data on intervention supervision

Supervision			
Healthcare profession	Modality	Frequency per week: Mean (SD)	Supervised sessions in %: Mean (SD)
Physio: 46% OT: 21% Physio, OT: 13% Multi: 11% Nurse: 3% Therapist: 3%	P: 76% R: 16% P, R: 9%	1,9 (1,1)	66 (36)

Note: Physio (physiotherapist), OT (occupational therapist), Multi (multidisciplinary team), P (presence), R (remote)

### Training specificity, home-based stimuli, teaching practices

Details on training specificity, home-based stimuli, teaching practices are provided in Table 4. Task specificity was adopted in almost all the studies (92%), while context specificity was implemented in 67% of the studies. Task specificity was implemented using ADLs and other functional activities, such as reaching and grasping for objects, walking, going up and down the stairs, and writing. The few studies that did not implement task specificity used muscle strengthening and static balancing exercises [24, 39, 80, 83]. While ADLs were always context specific, not all functional tasks were context specific. For instance, reaching and grasping was not always performed with everyday objects in daily contexts, but with standard objects [34, 60] while seated at a Table (71), and steps were performed up and down obstacles, not on the stairs of the house [67].

Regarding home-based stimuli, most of the studies did not provide any description about home modifications (65%) nor about the use of the variety of stimuli the home naturally provides (64%). Only 9% of the studies described home modifications, which in most cases involved the removal of hazards to increase safety [28, 78] and the provision of aids to support independence in movement [29, 48, 50, 73]. 22% of the studies utilized home-based variety of stimuli by encouraging participants to perform activities and movements, such as walking in different rooms of the house [66, 76], and upper-limb movements using the variety of objects present in the house [22, 54, 55, 65, 70, 73, 74, 85, 86].

Most of the studies (55%) did not provide information on the practices adopted to teach, instruct, and provide feedback on patients’ movements. 36% of the studies provided prescriptive instruction and feedback, while 9% of the studies provided open-ended instruction and feedback, promoting problem solving towards functional movements [64, 70], providing different movement strategies [35], encouraging to think about different ways of adapting movements [47], and with an emphasis on the goal of movement and not the movement itself [55].

**Table 4:** Aggregated data on training specificity, home-based stimuli, and teaching practices

Training specificity		Home-based stimuli		Teaching practice
Task-specific	Context-specific	Modifications	Variety	Prescriptive or open-ended
Yes: 92% No: 6% ND: 2%	Yes: 67% No: 21% ND: 12%	Yes: 9% No: 26% ND: 65%	Yes: 21% No: 15% ND: 64%	Prescriptive: 36% Open-ended: 9% ND: 55%

Note: ND (not described)

### Intervention design, individualization, task difficulty and progression, caregivers’ involvement

Details on Intervention design, individualization, task difficulty and progression, caregivers’ involvement are provided in Table 5. The intervention was for most of the studies (62%) designed by the healthcare professional, and the intervention was co-designed with the stroke survivors in 24% of the studies. The intervention was individualized in most studies (80%), while it adopted a one-size-fits-all approach in 11% of studies. Task difficulty and progression of challenge was for most of the studies (62%) individualized to each patient’s condition. Lastly, in more than half of the studies (55%), caregivers were involved in the intervention and their role varied, including supervision and support (33%), goal setting (2%), co-design (5%), and all the above (15%).

**Table 5:** Aggregated data on design, individualization, task difficulty and challenge, and caregivers’ involvement

Design	Individualization	Task difficulty and challenge	Caregivers’ involvement
**Co-designed or therapist-designed**	**Individualized or one-size-fits-all**		How they were involved
Co-designed: 24% Therapist-designed: 62% ND: 13%	Ind: 80% One-size-fits-all: 11% ND: 8%	Ind: 62% Pred: 5% ND: 36%	Supervision and support: 33% All steps: 15% Goal setting: 2% Co-design: 5% ND: 45%

Note: Ind (individualized), Pred (predefined), ND (not described)

## Discussion

### Main findings

This scoping review examined how home-based motor rehabilitation after stroke is designed and implemented, with a specific focus on training dose, task specificity, use of the home environment, instructional strategies, and the extent of personalisation and co-design of the intervention. While practices varied across settings and studies, several consistent gaps were identified. These include insufficient reporting and tailoring of training parameters, limited use of the home environment as an active rehabilitation site, underuse of theory derived evidence-based teaching strategies, and minimal involvement of caregivers and stroke survivors in intervention planning. Taken together, these findings suggest that the potential of home-based rehabilitation remains underutilised.

The studies included in this review mainly involved relatively young stroke survivors, with a mean age of 65, and most participants were in the chronic phase of their recovery, averaging two years post-stroke. Only a small number of studies (19% of the studies reporting the time since stroke) were conducted in the subacute phase of rehabilitation, i.e., fewer than three months post stroke. This reflects broader trends in stroke rehabilitation research, where interventions often target chronic populations. While home-based rehabilitation is increasingly recognised as a viable pathway following hospital discharge [88, 89], there is a growing consensus that greater attention should be given to the subacute phase, when the brain may be more responsive to interventions. The results of this review might have been different if more studies were conducted in the subacute phase where healthcare personnel and stroke survivors are likely to pay more attention to the rehabilitation strategies and conditions. This represents a direction for future research.

Regarding training dose, the average intervention duration was 13 weeks and aligns well with recommendations for long-term rehabilitation policy. However, the average completed weekly dose of 173 min appears insufficient when scrutinised against the same policy documents and guidelines, such as those from the World Health Organization [90] and the American Heart Association [91]. While the planned weekly dose across studies averaged 290 min, many studies failed to report completed dose, and only a small proportion provided direct measurements. Nevertheless, a subset of studies demonstrated that it was possible to maintain high levels of adherence even without continuous supervision.

Training specificity emerged as one of the best implemented components, with most studies incorporating task-specific activities such as reaching, walking, and writing. However, context specificity was less commonly applied, despite the assumption that practicing in the home naturally promotes real-world relevance. In practice, several studies used standardised objects or simulated tasks that were not integrated into participants’ actual environments nor aligned with their interests. Motor learning theory offers useful insights here: representative learning design emphasizes the importance of preserving relevant aspects of the task and context to enhance transfer of learning [7]. This means that not only the movements but also the surrounding environment and cognitive demands should resemble real-life conditions. Merely performing tasks in the home setting does not ensure context specificity unless the practice actively incorporates these essential features.

The review also revealed that the potential of the home environment as a rich source of rehabilitative stimuli remains largely untapped. Most studies did not describe environmental adaptations, and only a few made modifications aimed at improving safety or facilitating independence. These changes were typically static and not designed to evolve with the stroke survivor’s progress. Concepts from environmental enrichment (EE), originally developed in neuroscience and experimental biology [92], offer valuable guidance for transforming the home into a dynamic rehabilitation space. For instance, the different rooms of a patient’s home can be modified to constantly invite them to engage in repetitive, varied, and highly specific movement behaviours within their daily life. A recent review described a series of principles for designing physical and social environments that are relevant and tailored to each patient, providing complex, novel, and meaningful experiences within a patient’s daily life in the home [93]. Following these principles, the physical features of a home (spaces, size and location of objects) can be modified and tailored to each stroke survivor’s condition, inviting them to perform a variety of daily routine-related movements. Modifications occur regularly and slowly increase the challenge to stimulate adaptations of behaviour. The stroke survivor’s social circle (family members and friends) is involved in supporting this process, transforming home into a rehabilitation ecosystem that constantly provides survivor with rehabilitative stimuli. This has been a missed opportunity in current research and practice, and EE represents a major avenue for innovation in practice design.

An important yet frequently underreported aspect of rehabilitation studies is the method of instruction and teaching delivery. More than half of the reviewed studies lacked details on their instructional methods, and those that did include such information mainly relied on directive or prescriptive teaching strategies. Although structured guidance can offer clarity and consistency, it may also restrict opportunities for stroke survivors to engage in problem-solving, adapt their movements, and become more actively involved in their rehabilitation journey. A limited number of studies adopted more exploratory instructional techniques or feedback mechanisms that focused on movement goals rather than specific movement forms. These practices are aligned with motor learning principles that support variable and exploratory learning environments [94], which have been shown to disrupt ineffective motor patterns and promote the development of more flexible and resilient movement strategies in stroke survivors [95].

81% of studies within this review were tailored to participants’ needs and conditions, but only 23% of studies involved stroke survivors in co-designing their rehabilitation, and in many cases this information was not clearly reported. While the reported individualization aligns well with international calls for person-centred rehabilitation [96], co-design holds considerable potential to make rehabilitation more interactive and impactful by fostering greater relevance, user motivation, and a sense of ownership among participants [97]. For co-design solutions to be sustainable, stroke survivors need to be actively involved in tailoring solutions to their local context (home), sharing their experiences, and participating in decision-making, yet stroke survivors and caregivers are seldom involved in co-design beyond the acute phase, with limited focus on home rehabilitation uptake [98]. Further research emphasizes that co-design approaches often rely on preconceived intervention ideas and highlight that stroke survivors and caregivers value personalized stroke programs, demonstrating how preconceived design clash with the need for early involvement in tailored interventions [99]. This is particularly important when caregivers are involved, as their inclusion can help address the practical and emotional challenges of rehabilitation, promote consistency in daily routines, and strengthen the connection between the stroke individual, their environment, and their support system. Despite some practical challenges (e.g., limited time, insufficient training, and inadequate institutional support) may limit its adoption, co-design and caregiver involvement are crucial to make the home a truly interactive rehabilitation environment.

Caregiver involvement in stroke rehabilitation is often poorly reported and inconsistently applied. Despite their key role – especially with early discharge and community-based care – over half of studies lacked details on caregiver participation. When included, caregivers were typically limited to supervision or physical aid, with little input in planning or goal setting. This contrasts with strong evidence linking active caregiver engagement to better outcomes for stroke survivors and reduced burden on caregivers [100]. As healthcare increasingly shifts to families [101], it is crucial to recognize caregivers as essential partners, not just support, and adopt a more structured approach to integrating them into rehabilitation.

The limitations discussed thus far are quite surprising considering the wealth of publications and guidelines on applying principles of motor learning, neuroscience, and practice design to rehabilitation (e.g [8–10, 97]). What is probably lacking in the field for a full integration of evidence-based principles is an overarching framework that ties all the mentioned aspects together and provides practical guidance. There is a lesson to learn here from the motor learning field, where pedagogical frameworks, such as Nonlinear Pedagogy (NLP [102]), and Differential Learning [103], have been precisely developed to provide researchers and practitioners with a coherent set of linked principles and ideas on their application. For instance, NLP emphasises the importance of individual differences and adaptive learning environments, guiding the manipulation of task- and environment-related aspects, the delivery of appropriate instruction and feedback, while engaging relevant stakeholders in the process. In addition, the Cognitive Orientation to Daily Occupational Performance (CO-OP; [104]) represents a suitable framework to guide therapists towards a person-centred, goal-directed approach underpinned by cognitive and motor learning theories where the client is actively involved in the design of the rehabilitation pathway. Developing and embedding these pedagogical approaches into clinician training could be the way forward to bridge the gap between theoretical insights and day-to-day clinical practice, ultimately contributing to more personalized and effective home-based rehabilitation programs.

This review provides a series of directions for future research. Existing meta-analyses show contrasting results on the effect of home-based rehabilitation on a variety of outcomes (e.g [18, 19]). Integrating the aspects considered in this review into future meta-analyses can facilitate a parsimonious extrapolation of the effects of home-based rehabilitation, teasing out the moderating role of practice conditions. Further, to test all the suggestions made throughout the discussion, a future direction is to conduct a trial comparing standards versus enriched, co-designed home environments.

## Conclusion

This scoping review showed that while home-based motor rehabilitation offers significant potential, key components remain insufficiently developed. Common limitations include inadequate training dosage, limited contextual adaptation to the home environment, and a lack of integration of learning principles grounded in EE and motor learning science. Instructional methods tend to be overly prescriptive, co-design strategies are applied inconsistently, and caregiver engagement is often underutilised. To address this gap between theory and practice, future research and clinical applications should prioritise early-phase interventions with adequate intensity, and develop relevant pedagogical frameworks, such as NLP, to guide home modifications and instructional methods, supporting progressive and engaging practice, and fully adopt co-designed, person-centred approaches that meaningfully involve family caregivers. These steps are essential to making home-based rehabilitation a more effective, relevant, and sustainable approach to post-stroke recovery.

Name: Additional file 1

File format: Word document. docx

Title of data: PRISMA-ScR checklist

Description of data: This file aligns this manuscript with the PRISMA-ScR checklist

## Supplementary Information

Below is the link to the electronic supplementary material.

Supplementary Material 1: Name: Additional file 1. File format: Word document .docx. Title of data: Inclusion and exclusion criteria. Description of data: This file reports the full list of inclusion and exclusion criteria

Supplementary Material 2: Name: Additional file 2. File format: Word document .docx. Title of data: Search strings. Description of data: This file presents the search strings tailored to each database

Supplementary Material 3: Name: Additional file 3. File format: Excel document. xlsx. Title of data: Dataset. Description of data: This file presents the dataset of information extracted from all the included studies

Supplementary Material 4: Name: Additional file 4. File format: Word document. docxTitle of data: PRISMA-ScR checklist Description of data: This file aligns this manuscript with the PRISMA-ScR checklist

## Data Availability

The full set of data extracted from the studies is provided in additional file 3.

## Abbreviations

ADL: Activities of daily living
PRISMA: Preferred Reporting Items for Systematic reviews and Meta-Analyses
EE: Environmental enrichment
NLP: Nonlinear pedagogy
