# Supplementary material for: Strengths, limitations, and way forward of home-based rehabilitation practices after stroke: a scoping review
Source: BMC Health Serv Res. 2026 Feb 25;26:447. doi: 10.1186/s12913-026-14139-4 (PMC13040734; doi:10.1186/s12913-026-14139-4)
Supplement: Supplementary file 1 — Supplementary Material 1: Name: Additional file 1. File format: Word document .docx. Title of data: Inclusion and exclusion criteria. Description of data: This file reports the full list of inclusion and exclusion criteria [file 12913_2026_14139_MOESM1_ESM.docx]

| **Inclusion criteria** | **Exclusion criteria** |
| --- | --- |
| Population: stroke survivors | Other populations |
| Intervention: Studies that conducted (or planned to conduct) a motor rehabilitation intervention in the patient’s home after stroke, led by a healthcare professional (physiotherapist, occupational therapist, or nurse) | Studies that implemented a home-based intervention as control group, included technology-based interventions as a focus, or lacked a description of the intervention |
| Peer-reviewed journal articles, published in English | Other publications and other languages |
