# Supplementary material for: Strengths, limitations, and way forward of home-based rehabilitation practices after stroke: a scoping review
Source: BMC Health Serv Res. 2026 Feb 25;26:447. doi: 10.1186/s12913-026-14139-4 (PMC13040734; doi:10.1186/s12913-026-14139-4)
Supplement: Supplementary file 2 — Supplementary Material 2: Name: Additional file 2. File format: Word document .docx. Title of data: Search strings. Description of data: This file presents the search strings tailored to each database [file 12913_2026_14139_MOESM2_ESM.docx]

**PubMed and CINAHL:**

(stroke OR cerebrovascular) AND (home OR "home-based" OR "home based" OR "home environment" OR residential OR domiciliary OR home-delivered OR domestic OR home-centered OR home-centred OR "home care services" OR “supported discharge”) AND ("stroke rehabilitation" OR rehabilitation OR treatment OR rehabilitative OR therapy OR recovery or “task specific training” or “task oriented training”) AND ("physical therapists" OR therapist OR therapist-led OR "therapist led" OR care-supported OR "care supported" OR physiotherapist OR nurses OR nurse OR specialist) AND (trial OR pilot OR experiment* OR intervention) NOT "systematic review"

**PsycInfo:**

(((stroke or cerebrovascular) and (home or "home-based" or "home based" or "home environment" or residential or domiciliary or home-delivered or domestic or home-centered or home-centred or "home care services" or "supported discharge") and ("stroke rehabilitation" or rehabilitation or treatment or rehabilitative or therapy or recovery or “task specific training” or “task oriented training”) and ("physical therapists" or therapist or therapist-led or "therapist led" or care-supported or "care supported" or physiotherapist or nurses or nurse or specialist) and (trial or pilot or experiment* or intervention)) not "systematic review").ab,hw,id,mh,mf,nl,ot,sh,ti,tw.

**Medline:**

(((stroke or cerebrovascular) and (home or "home-based" or "home based" or "home environment" or residential or domiciliary or home-delivered or domestic or home-centered or home-centred or "home care services" or "supported discharge") and ("stroke rehabilitation" or rehabilitation or treatment or rehabilitative or therapy or recovery or “task specific training” or “task oriented training”) and ("physical therapists" or therapist or therapist-led or "therapist led" or care-supported or "care supported" or physiotherapist or nurses or nurse or specialist) and (trial or pilot or experiment* or intervention)) not "systematic review").af.

**Web of Science:**

stroke OR cerebrovascular (Topic) and home OR "home-based" OR "home based" OR "home environment" OR residential OR domiciliary OR home-delivered OR domestic OR home-centered OR home-centred OR "home care services" OR “supported discharge” (Topic) and "stroke rehabilitation" OR rehabilitation OR treatment OR rehabilitative OR therapy OR recovery or “task specific training” or “task oriented training” (Topic) and "physical therapists" OR therapist OR therapist-led OR "therapist led" OR care-supported OR "care supported" OR physiotherapist OR nurses OR nurse OR specialist (Topic) and trial OR pilot OR experiment* OR intervention (Topic) not "systematic review" (Topic)
